# The Role of Hydrodynamic Processes on Anchovy Eggs and Larvae Distribution in the Sicily Channel (Mediterranean Sea): A Case Study for the 2004 Data Set

**DOI:** 10.1371/journal.pone.0123213

**Published:** 2015-04-27

**Authors:** Federico Falcini, Luigi Palatella, Angela Cuttitta, Bruno Buongiorno Nardelli, Guglielmo Lacorata, Alessandra S. Lanotte, Bernardino Patti, Rosalia Santoleri

**Affiliations:** 1 Istituto di Scienze dell’ Atmosfera e del Clima, Consiglio Nazionale delle Ricerche, Rome, Italy; 2 Istituto di Scienze dell’ Atmosfera e del Clima, Consiglio Nazionale delle Ricerche, Lecce, Italy; 3 Istituto per l’Ambiente Marino Costiero, Consiglio Nazionale delle Ricerche, Capo Granitola (TP), Italy; 4 Istituto per l’Ambiente Marino Costiero, Consiglio Nazionale delle Ricerche, Napoli, Italy; Deakin University, AUSTRALIA

## Abstract

Knowledge of the link between ocean hydrodynamics and distribution of small pelagic fish species is fundamental for the sustainable management of fishery resources. Both commercial and scientific communities are indeed seeking to provide services that could “connect the dots” among *in situ* and remote observations, numerical ocean modelling, and fisheries. In the Mediterranean Sea and, in particular, in the Sicily Channel the reproductive strategy of the European Anchovy (*Engraulis encrasicolus*) is strongly influenced by the oceanographic patterns, which are often visible in sea surface temperature satellite data. Based on these experimental evidences, we propose here a more general approach where the role of ocean currents, wind effects, and mesoscale activity are tied together. To investigate how these features affect anchovy larvae distribution, we pair ichthyoplankton observations to a wide remote sensing data set, and to Lagrangian numerical simulations for larval transport. Our analysis shows that while the wind-induced coastal current is able to transport anchovy larvae from spawning areas to the recruiting area off the Sicilian south-eastern tip, significant cross-shore transport due to the combination of strong northwesterly mistral winds and topographic effects delivers larvae away from the coastal conveyor belt. We then use a potential vorticity approach to describe the occurrence of larvae cross-shore transport. We conclude that monitoring and quantifying the upwelling on the southern Sicilian coast during the spawning season allows to estimate the cross-shore transport of larvae and the consequent decrease of individuals within the recruiting area.

## Introduction

Since the ancient times, human activities have had a major impact on the life cycle and reproductive behaviour of a number of fish species [1]. Overfishing, indeed, has been recognized as the primary cause of species depletion and/or individuals’ size reduction [2], [3]. In addition, the industrial era led to a “recent” environmental collapse by impacting the ecosystem that supports fish species [2]. Besides the anthropogenic issues, ocean processes also play a significant role on productive capacity [4], [5]. “Food” concentration and availability, for instance, are modulated by oceanographic structures that have a crucial effect on the fate of several species, especially during their larval and juvenile stages [6], [7]. Moreover, hydrographic and dynamical properties of the marine environment may affect the recruitment of small pelagic fish species and characterize their spawning habitat [8–13]. This is particularly evident in the Mediterranean Sea, a semi-enclosed basin whose environmental characteristics are sensitive to both large scale and local effects (e.g., winds, topography). Here, fluctuations of fish populations are not exclusively linked to fishing activity, but rather they are significantly affected by ocean conditions [14], [15]. As an example, in the Gulf of Lions (NW Mediterranean) river inputs and wind mixing were found to favour fish productivity [15]. An additional example is given by the pattern of adult turtles dispersion from the breeding area, which reflects the extent of passive dispersion that would be experienced by hatchlings and stresses to role of ocean currents on the selection of foraging sites used by adult sea turtles [16].

Among the most important economic resources for Mediterranean fisheries, the European Anchovy (*Engraulis encrasicolus*, Linnaeus, 1758) and more in general small pelagic fish species, also called forage fish, play a fundamental ecological role due to their intermediate position in the marine trophic web. These small, plankton-feeding fish species have a major impact on the trophic dynamics due to their top-down control of zooplankton and/or bottom-up control of larger predators (fish, marine birds and marine mammals). They are dominated by one or a few species, and characterized by extremely variable size, especially under intensive exploitation and/or unfavourable environmental conditions. This is confirmed by the fact that European Anchovy distribution along the Mediterranean coasts shows a set of independent stock units [17], a patchy distribution that points out the strong environmental control on spawning areas and rates, recruiting, and landing.

In particular, the Sicilian Channel Anchovy adapted its reproductive strategy to the surface circulation in the area [18–20]. The Sicily Channel is mainly characterized by a meandering surface current, the Atlantic Ionian Stream (AIS; [21]), which transports the surface waters of Atlantic origin eastwards. The climatological pattern of the AIS encircles two cyclonic vortices over the Adventure Bank and off Cape Passero, i.e., the Adventure Bank Vortex (ABV) and the Ionian Shelf Break Vortex (IBV), respectively, and it describes a pronounced anticyclonic meander in between, i.e., the Maltese Channel Crest (MCC) [21–23]. In the intermediate layers, an important current transports the warm and salty Levantine waters across the Sicily channel, subject to strong topographical constraints and intense mixing along its flow towards the western sub-basins [24]. This entire system is thus characterized by a complex physical dynamics, combining large scale thermohaline circulation and associated instabilities with local processes (such as wind-driven upwelling and topographical effects along the coasts of Sicily) that result in an intense variability at both (sub)mesoscale and sub-basin scales (e.g. [25–29]). In fact, wind forcing plays an important role in modulating the oceanographic structures described above. The northwesterly Mistral wind, in particular, was recognized to enhance both the AIS intensity and its meandering structure during summer, because of the role of upwelling in the ABV and IBV regions [30], [31].

Hydrographic features have clear influence on the early stages of the life cycle of the Sicily Channel anchovy [18]-[20], [32]. Spawning and egg transport efficiency have a large and rapid impact on fishery since the anchovy is a short lived species (3–4yrs). This also reflects on anchovy landings, which highly depends on annual recruitment. In particular, the offshore displacement of the AIS would account for the low spawning of anchovy observed in 1999 and 2000 [19]. According to Garcìa Lafuente et al. [20], anchovy spawning preferably occurs in the area where the AIS approaches the coast. As mentioned above, the AIS on- or offshore displacement leads to the appearance/disappearance of the cyclonic vortices (ABV and IBV)—which however are not present all year round and are subject to intense seasonal variability—and the location of the MCC, which represents a critical factor in determining the position of the main spawning grounds. All these patterns modify the temperature regime of the surface waters in the Sicilian anchovy habitat and represent the main source of environmental variability impacting the anchovy spawning ground [32–34].

Apart from determining the spawning rates and areas along the coast, the AIS pattern also affects anchovy eggs and larvae transport processes towards the Sicilian south-east end, off Cape Passero, where larvae are retained in a frontal structure that originates from the meeting of AIS and Ionian Sea water masses [20]. However, the AIS alone cannot explain the complex dynamics that characterize anchovy eggs and larvae path and fate. Indeed, additional large, mesoscale and submesocale activities such as upwelling-induced coastal currents, cold filaments, and topographic induced baroclinic instabilities may affect the journey of these biological tracers, causing depletion in the nursery areas. Fluctuations on the efficiency of transport phenomena in connecting spawning and nursery areas to each other have been recently investigated by means of a well-designed Lagrangian approach [35], which can be employed in a multivariate context for investigating population dynamics along with other environmental parameters.

The goal of our work is to provide a synthesis of processes and hydrographic patterns that affected the anchovy eggs and larvae transport and distribution, as recognized from the ichthyoplanktonic survey ANSIC carried out in June-July 2004 in the Sicily Channel. For such a case study we pair measurements of anchovy eggs concentration, anchovy larvae length, age, and distribution with remote sensing data such as sea surface temperature (SST), chlorophyll (Chl), light attenuation coefficients (Kd490), wind field, and altimetric data. A multivariate Empirical Orthogonal Function decomposition (EOF) technique is also used to further examine the relationships between observed oceanographic patterns, environmental variables, and larvae distribution. Finally, to better investigate transport phenomena, we make use of Lagrangian simulations and theoretical analyses that allow to correctly frame observed oceanographic phenomena

## Data and Methods

**Fig 1:**
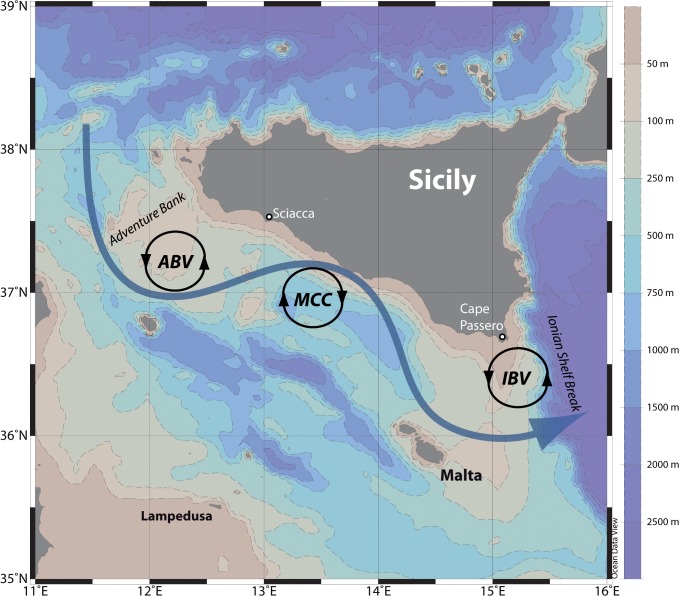
The Sicily Channel. Bathymetry and the climatologic hydrographic setting of the Sicily Channel. Blue arrowed curve: AIS path; black closed lines: ABV, MCC, and IBV gyres (see text). The geographical locations cited in this work are also indicated.

Our case study area is located off the southern Sicilian coast (Fig 1) in the Sicily Channel (Mediterranean Sea).

### *In situ* data

Biological samples were collected during the oceanographic surveys ANSIC 2004 on board the R/V *Urania* of the Italian National Research Council from 18 June to 7 July 2004. Sample stations constitute a regular grid (1/10° × 1/10° along the continental shelf, and 1/5° × 1/5° offshore) made of transects that were perpendicular to the coast. Ichthyoplanktonic samples were obtained using Bongo 40 and 60 plankton net [36] equipped with two flow meters, with oblique tows deployed at a speed of 2 knots (20 m/min), using a 200 μm mesh size net for both sides of the frame. The nets were hauled from the bottom to the surface or from 100 m to the surface where the depth was more than 100 m. Plankton samples were immediately fixed after collection, preserved in a 4% buffered formaldehyde and seawater solution and then transported to the land based laboratory where they were sorted for further analyses. Fish larvae have been recognized with the taxonomic keys as in [34], [37–43].

To measure the growth of larval fish we have studied otoliths that are enabled to reconstruct the responses of fishes to their physical and biological environments. It is well known that the microstructure of otoliths contains lamellar structures that accurately record yearly and daily growth. We extracted both otoliths from the otic room using micro needles. Otolith extracts were photographed and the images stored. We used "Image pro plus" in order to capture otolith images from the microscopes that can be more easily interpreted than the original [37–39]. The study was carried out on data collected in this area without any restriction. We received the necessary permissions for working at sea in National waters by the Italian Coastal Guards for the Strait of Sicily. We state that no specific permissions were required for the surveyed area in the Strait of Sicily and, since the involved Institute (IAMC-CNR) were charged by their Ministries to carry out the data collection in the framework of the work programme of research project AMECO (Alice MEditerranea Crescita ed Oceanografia) funded by the Italian “Ministero dell’Università e della Ricerca Scientifica e Tecnologica”. We confirm that the field studies did not involve endangered or protected species.

### Remote sensing data

Remote sensing data were obtained from different sensors and interpolating processes (S1 Table). We consider, in particular, daily Optimally Interpolated Sea surface SST data [44], Kinetic Energy (KE) estimated from AVISO (Archiving Validation and Interpretation of Satellite Oceanographic Data) altimetry products, daily chlorophyll (Chl) and diffuse attenuation coefficient (K490) data from SeaWiFS. This last coefficient can be considered as a proxy for concentration of particles and might serve to model light propagation in the water column in the visible spectral range, and it is thus of potential use for physical and ecosystem numerical models [45].

Moreover, we used ocean surface 6-hourly wind data (${\vec{U}}_{wind}$), derived under the Cross-Calibrated Multi-Platform project (S1 Table), to evaluate: wind stress ($\vec{\tau }=\rho_{air}C_{d}\left|{\vec{U}}_{wind}\right|{\vec{U}}_{wind}$), where *ρ*_*air*_ is the air density and the dimensionless friction coefficient *C*_*d*_ = 0.0012 for $0<\left|{\vec{U}}_{wind}\right|<11$ m/s and *C*_*d*_ = 0.00049 for $\left|{\vec{U}}_{wind}\right|\geq 11$ [46], [47]; Ekman transport $\vec{M}={\left(\rho_{water}f\right)}^{-1}\vec{\tau }\times \hat{k}$ [48], where *ρ*_*water*_ *ρ*_*water*_ is the water density, *f* the Coriolis parameter, and $\hat{k}$ is the vertical unit vector; Ekman layer $h_{EK}=e_{k}/f{\left(\left|\vec{\tau }\right|/\rho_{water}\right)}^{\frac{1}{2}}$, where *e*_*k*_ = 0.4 is a dimensionless coefficient [49].

### Data comparison

**Fig 2:**
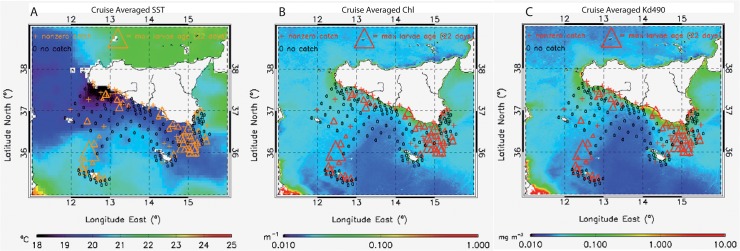
Cruise averaged satellite products and anchovy larvae distribution. Ichthyoplanktonic sampling stations of the ANSIC 2004 survey. Triangles indicate larvae ages inferred from otoliths, ‘o’ indicates no catch, ‘+’ indicates stations with non-zero catch, where the otoliths were not measured. These stations are superimposed to cruise-averaged maps of satellite (A) SST, (B) Chl, and (C) Kd490. See also anchovy larvae concentrations in S2 Fig.

We performed daily, weekly and monthly analyses, pairing the ichthyoplanktonic data to the satellite SST, Chl and Kd490 data. We also performed a cruise average analysis of the satellite daily data (Fig 2); cruise average spatial maps of each environmental parameter were superimposed to the entire ichthyoplanktonic data set. This allowed to first recognize the main patterns and relations between the biological and environmental datasets.

### Empirical Orthogonal Function decomposition

The multivariate EOF analysis (also known as Extended EOF or Principal Component Analysis, PCA) is a technique to identify the patterns that maximize the variance of a multivariate state vector that is made of a given set of parameters. EOFs are obtained by diagonalizing the covariance matrix obtained from available samples of the multivariate state vector and thus allow to describe each sample as the linear combination of modes (the eigenvectors), a few of which are generally able to reproduce most of the observed variance. Here we built the state vector by putting together normalized larval concentration, Chl, SST, KE, and Ekman transport. Though Gaussian distributions are not required by EOF, as no higher order statistics beyond variance are needed, the technique is more efficient in representing multivariate normally distributed data. Consequently, we preliminary log-transformed larval concentration, and Chl to get less skewed distributions.

As we are here dealing with samples that are collected in different locations, with no temporal repetition, the EOF analysis will provide information on the spatial variability associated with the multivariate state vector, not necessarily related to specific oceanographic or ecological processes, but possibly reflecting common multivariate patterns which could help to identify the distribution of larvae in terms of environmental conditions and oceanographic processes. With respect to this aim, it will not be important to analyse the patterns that explain the highest percentage of the multivariate state vector, but rather to concentrate on those modes that explain most of the larvae variability.

### Lagrangian modelling

We used Lagrangian simulations to depict anchovy eggs and larvae transport in the Sicily Channel during the spawning season (i.e., June—July) of 2004 [35]. As Eulerian input for the Lagrangian model we used velocity fields provided by the Mediterranean Sea Forecasting System (MFS) model [50], which is based on the primitive Navier-Stokes equations with the Boussinesq, hydrostatic, and incompressible approximations as solved through the Nucleus for European Modelling of the Ocean (NEMO) code. The MFS solutions are corrected through a variational assimilation of temperature and salinity vertical profiles and along track satellite sea level anomaly observations (based on a 3D-VAR scheme). Horizontal and vertical resolutions of the MFS model are: 1/16° × 1/16° in the horizontal directions (≈ 6.5 km), and 72 vertical levels. The horizontal eddy viscosity and eddy diffusivity are taken as constant, and equal to 5 × 10^9^ m^4^/s and 3 × 10^9^ m^4^/s, respectively. Vertical diffusivity and viscosity are both a function of the Richardson number [50]. For this study, we considered daily snapshots from the model analysis of the zonal and meridional components of the velocity fields, covering the summer months from June to September 2004. Transport and diffusion properties were then inferred from simulations of Lagrangian trajectory evolution. Dynamics on scales poorly, or not at all, resolved by the MFS model, i.e. the horizontal sub-mesoscale range and the vertical mixed layer range, is parameterized in terms of kinematic models, i.e. deterministic, nonlinear, time-dependent, incompressible, analytical velocity fields that exploit Lagrangian chaos [51] as the primary mechanism of tracer dispersion. In particular, we use 2D and 3D lattices of non-steady convective cells [35], [52], [53], which periodically oscillate around their mean positions. Normally, once these models have been suitably set up to generate chaotic trajectories, they succeed in simulating tracer transport and diffusion in various regimes, with characteristic properties that can be controlled and adapted to specific cases. This, for instance, allows to restore (in a statistical sense) small-scale dispersion rates otherwise underestimated by general circulation models [54].

## Results from Ichthyoplanktonic and Satellite Data

The ANSIC 2004 ichthyoplanktonic data confirm that anchovy spawning preferably occurs in the area where the AIS approaches the coast (MCC) [20], [55]. Indeed, eggs are mainly concentrated along the southern Sicilian coastal regions washed by the AIS, which is easily detected by the cruise averaged SST pattern (S1A Fig).

Larvae are then transported south-eastwards, along the Sicilian coasts, and they will be retained off Cape Passero (Fig 2 and S1B Fig). This is an important recruiting area: due to the presence of the IBV and, in particular, of the Maltese Front, which is given by the contrast of the exiting Atlantic waters with the warmer Ionian Sea, anchovy are here able to grow in good environmental and biological conditions [31]. Indeed, the highest concentration of mature (10–20 days) larvae is recorded off Cape Passero within the IBV, where high values of chlorophyll and absorption coefficient are seen from remote sensing observations (Fig 2).

These results, in general, suggest that the use of remote sensing data provides a fairly good tool for inferring and predicting the statistical pattern of fish and fish larvae habitats **[** 56]. Due to the characteristic sea surface dynamics of the Sicily Channel, SST and/or Ocean Color products can indeed mark advective paths and retention areas. However, these observations alone cannot be used to infer transport phenomena and thus to understand the actual dynamics of fish larvae (and other observables related to fishery). Indeed we are here mainly focused on an approach which, rather than limiting to recognize correlations between the presence of larvae and remote sensing data, seeks to understand larvae distribution and fate from known oceanographic processes. This particular aim arises from the unexpected presence of some anchovy larvae around the island of Lampedusa within a slightly cold, nutrient rich strip of water (Fig 2).

**Fig 3:**
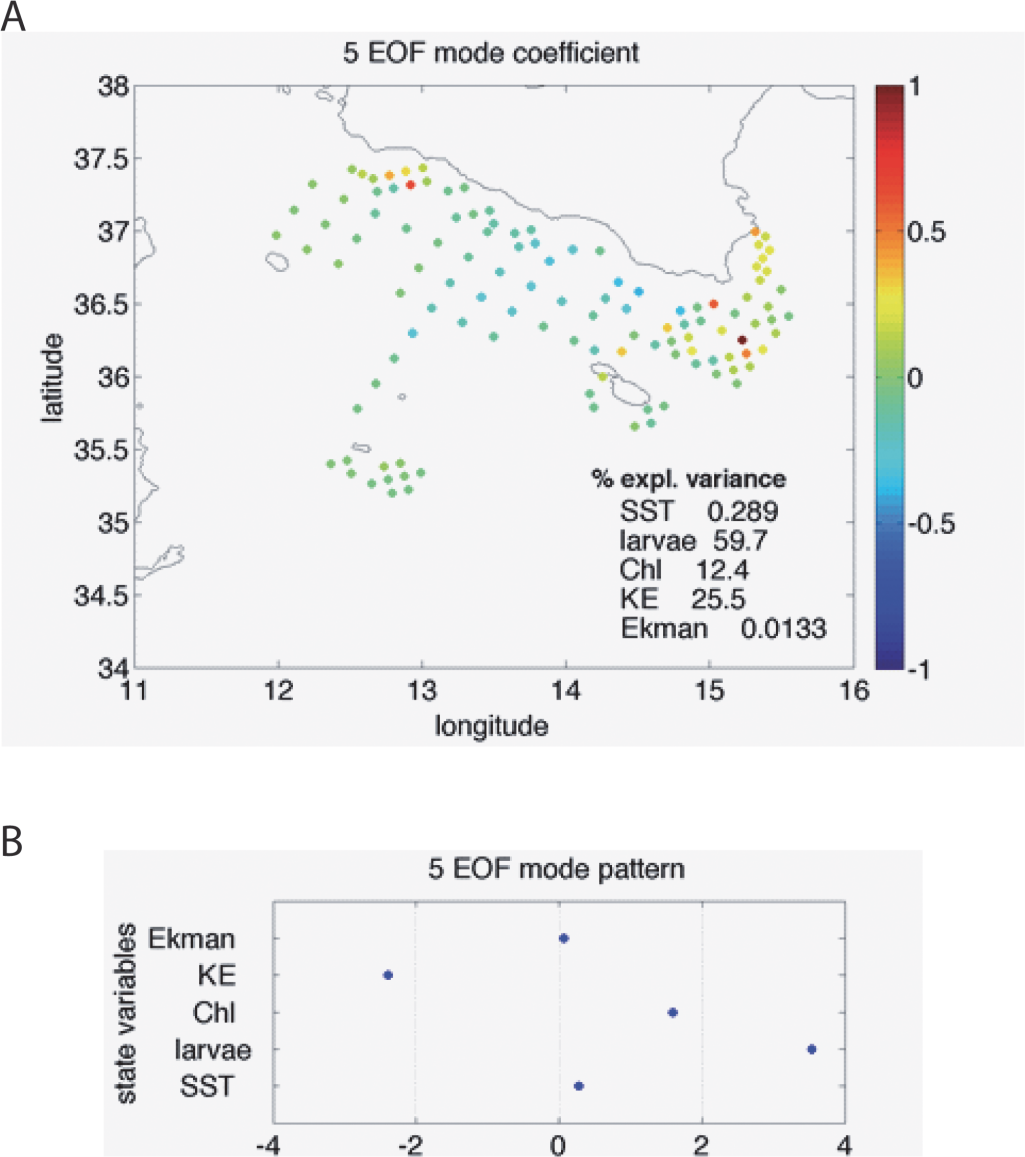
Spatial coefficients (A) and multivariate pattern (B) obtained from the 5th multivariate EOF. This mode highlights the areas where high Larvae concentrations are associated with relatively high chlorophyll and low kinetic energy, thus mainly identifying retention areas close to Sciacca and Cape Passero.

**Fig 4:**
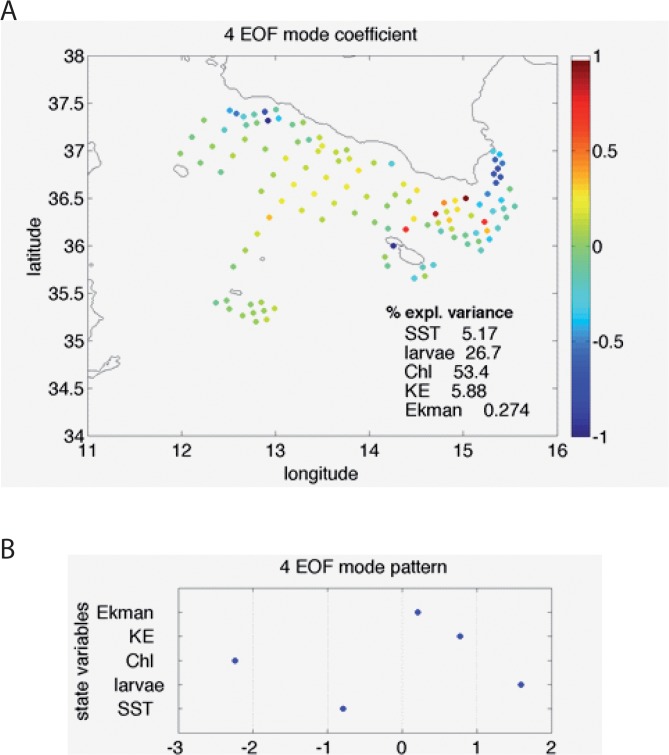
Spatial coefficients (A) and multivariate pattern (B) obtained from the 4th multivariate EOF. This mode highlights the areas where high Larvae concentrations are associated with relatively low chlorophyll, high kinetic energy, and colder waters, namely the central part of the Channel and off the South-Eastern coasts of Sicily.

In fact, the EOF decomposition displays two main modes accounting for larvae distribution (S2 Fig, orange curve). The spatial pattern associated with Mode 5, explaining about 60% of the larvae concentration variance, clearly identifies the retention zones close to the Sicilian coast and off Cape Passero (Fig 3A). Looking at corresponding multivariate pattern (Fig 3B), it is evident how this mode reflects the environmental conditions that are most favourable for anchovy recruitment: low kinetic energy and high chlorophyll. Conversely, Mode 4, which accounts for slightly less than 27% of larvae variance (S2 Fig), displays positive anomalies offshore, in correspondence of negative Chl and SST anomalies, with positive KE (Fig 4). For such a pattern we can hypothesize the occurrence of a cross-shore transport process, originating from strong wind event that would remove larvae from the major, coastal conveyor belt [35].

**Fig 5:**
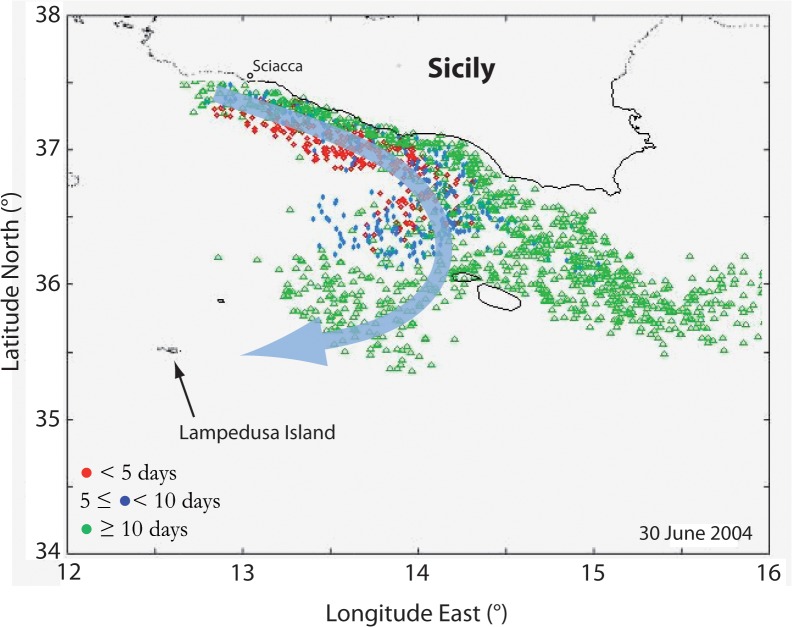
Lagrangian simulation for anchovy eggs and larvae transport. The 30 June 2004 snapshot of the Lagrangian model run of larval transport [35] shows the occurrence of an anti-cyclonic branch (thick arrowed line) that separates from the main coastal pathway and delivers larvae toward Lampedusa Island. The simulated spawning area is off Sciacca and colours represent different larvae age ranges.

Our hypothesis is confirmed by the Lagrangian simulation for the period June-July 2004 (S1 Movie), which shows an offshore branch of the coastal current that flows anti-cyclonically towards Lampedusa (Fig 5). We also recognize that this flow separation occurs upstream, i.e. west of Malta, where the bathymetry is characterized by an increase in curvature of the about 100–250 m isobaths (Fig 1). Such a circulation, hypothesized but not observed by Garcìa-Lafuente et al. [20], suggests an effective role of winds and/or topographic effects that may trigger anchovy cross-shore transport processes during particular conditions [25], [30].

**Fig 6:**
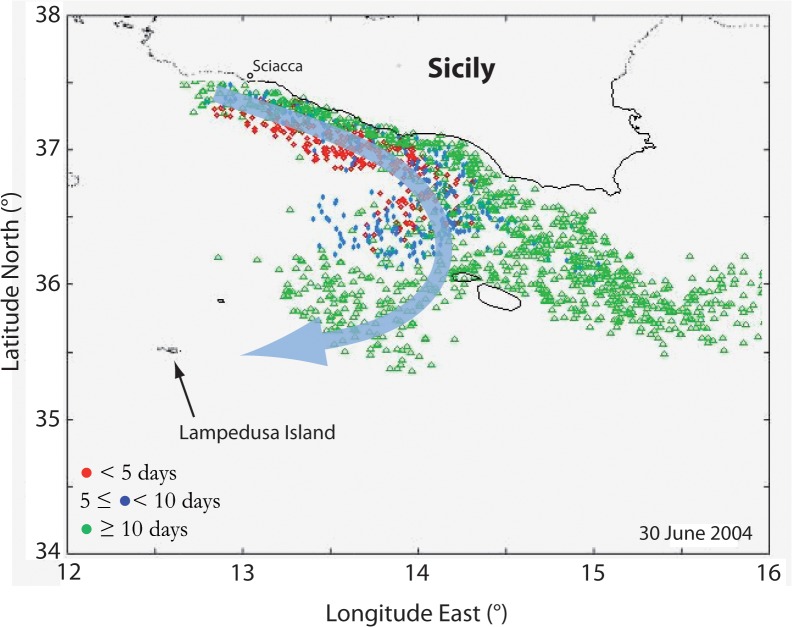
Coastal upwelling along the Sicilian coast (30 June 2004). It shows the formation of an upwelled coastal pattern marked by (A) a strong satellite SST gradient along the southern Sicilian shoreline and (B) a strong Ekman offshore transport (see also S2 Fig).

The role of wind forcing and upwelling is therefore analysed by using satellite daily SST and wind data (Fig 6 and S3 Fig). Our results reveal a strong upwelling off Sciacca during the first week of the sampling survey and the consequent formation of an alongshore SST pattern (i.e., cold waters along the Sicilian coast) that reasonably indicates an upwelling-induced alongshore coastal flow (S3A Fig). Upwelling is also confirmed by the strong Ekman transport that occurs around June 30^th^ (Fig 6). This geostrophic flow might therefore experience a topographic-induced instability where the sea bottom decreases (i.e., upstream of Malta). The consequence of such an instability may give rise to a flow separation and thus to a significant cross-shore transport, which coincides with what scalar parameter (SST, Chl) observations suggest.

### The Potential Vorticity Model for Coastal Hydraulics

Our observations and hypotheses bring us to seek a physically-based, analytical synthesis that can “connect the dots” among upwelling, coastal transport, topographic changes (i.e., coastal curvature), and flow separation. As suggested by *Pratt and Whitehead* [57], the analysis of the equations of motion in a coast-following coordinate system may represent a good start for the physical interpretation of our findings.

Being *s* and *n* the along- and off-shore directions, and *v* and *u* the corresponding velocity components (S4 Fig), we can write the equations of motion as [57]

$$\frac{\partial v}{\partial t}+\frac{r}{r+n}v\frac{\partial v}{\partial s}+u\frac{\partial v}{\partial n}+\frac{uv}{r+n}-fu=-g\frac{r}{r+n}\frac{\partial \left(d+h\right)}{\partial s}$$ (1)

$$\frac{\partial u}{\partial t}+u\frac{\partial u}{\partial n}+\frac{r}{r+n}v\frac{\partial u}{\partial s}-\frac{v^{2}}{r+n}+fv=-g\frac{\partial \left(d+h\right)}{\partial n}$$ (2)

$$\frac{r+n}{r}\frac{\partial d}{\partial t}+\frac{\partial \left(vd\right)}{\partial s}+\frac{\partial }{\partial n}\left[\frac{r+n}{r}ud\right]=0$$ (3)

Eqs (1)–(3) represent, respectively, the momentum balance along *s* and *n*, and the continuity of a surface coastal current of thickness *d*, where the radius of curvature *r*(*s*) of the coastline (or of a chosen isobath) is considered positive if the coast curves to the right, stream-wise (i.e., in the direction of increasing *s*). We note that in such a cylindrical frame the origin of the system is at a distance *r*(*s*) from the coast so that *D* = *r*(*s*) + *n* is a constant (S4 Fig). In (1)–(3) *h*(*s*,*n*) is the topography, and *g* gravity.

By scaling the dimensional variables as

$$r=r_{0}r',\;d=Dd',\;(n,s)=(Wn',Ls'),\;(v,u)=(Vv',Uu'),\;t=L/{\left(gD\right)}^{1/2}t'$$ (4)

and by using the relations *U* = *VW*/*L*, W = (*gD*)^1/2^/*f*, and *V* = (*gD*)^1/2^, the dimensionless forms of (1)–(3) are:

$$\frac{\partial v}{\partial t}+\frac{r}{r+\frac{W}{r_{0}}n}v\frac{\partial v}{\partial s}+u\frac{\partial v}{\partial n}+\frac{W}{r_{0}}\frac{uv}{r+\frac{W}{r_{0}}n}-u=-\frac{r}{r+\frac{W}{r_{0}}n}\frac{\partial \left(d+h\right)}{\partial s},$$ (5)

$$\frac{W^{2}}{L^{2}}\left[\frac{\partial u}{\partial t}+u\frac{\partial u}{\partial n}+\frac{r}{r+\frac{W}{r_{0}}n}v\frac{\partial u}{\partial s}\right]-\frac{W}{r_{0}}\frac{v^{2}}{\left(r+\frac{W}{r_{0}}n\right)}+v=-\frac{\partial \left(d+h\right)}{\partial n},$$ (6)

$$\frac{r+\frac{W}{r_{0}}n}{r}\frac{\partial d}{\partial t}+\frac{\partial \left(vd\right)}{\partial s}+\frac{\partial }{\partial n}\left[\frac{r+\frac{W}{r_{0}}n}{r}ud\right]=0,$$ (7)

where, for sake of simplicity, we omit the prime symbol on all the dimensionless variables *r*, *d*, *n*, *s*, *u*, and *v*. Let us also point out that *W* and *r*_0_ are the characteristic width of the coastal current and the characteristic radius of curvature of the coast, respectively.

In particular, the dimensional analysis of (6) tells us that, for a narrow current (i.e., *W*/*L* << 1) flowing along a gradually varying coast, the cross-stream momentum balance (2) reduces to a geostrophic component plus the effect of curvature [57]:

$$-\frac{W}{r_{0}}\frac{v^{2}}{\left(r+\frac{W}{r_{0}}n\right)}+v=-\frac{\partial \left(d+h\right)}{\partial n}\;\Rightarrow \;-\frac{v^{2}}{r}+v=-\frac{\partial \left(d+h\right)}{\partial n},$$ (8)

where advective terms of *O*(*W* / *L*)^2^ are neglected. We also considered that the local (dimensional) radius of curvature *r*(*s*) + *n* (S4 Fig) is approximated as *r*(*s*).

To describe the coastal transport dynamics in term of flow spatial characteristics and bathymetric curvature we can now make use of the system (1)–(3) or, alternatively, the approximated cross-stream momentum balance (8) instead of (2). By cross-differentiating (1) and (2), and with the use of (3) one obtains the potential vorticity (PV, denoted by *q*) conservation [57], [58]:

$$\left[\frac{\partial }{\partial t}+u\frac{\partial }{\partial n}+\frac{r}{r+n}v\frac{\partial }{\partial s}\right]q=0,$$ (9)

where

$$q_{non-lin}\equiv \frac{f+\frac{r}{r+n}\frac{\partial u}{\partial s}-\frac{\partial v}{\partial n}-\frac{v}{r+n}}{d}=const.$$ (10)

The PV in (10) represents a scalar quantity that is conserved in adiabatic and frictionless conditions and describes the rotational properties of the flow (i.e., non-linear local rotation ($\frac{\partial u}{\partial s}-\frac{\partial v}{\partial n}$) and curvature ($\frac{v}{r+n}$) of the flow), depending on the stretching of the water column *d* which, in turn, depends on the topographic constraint.

Alternatively, instead of the cross-stream momentum balance (2) one can use the approximate Eq (8) for a narrow current (i.e., *W*/*L* << 1), flowing along a gradually varying coast. The consequent PV equation for this approximations is

$$q_{lin}\equiv \frac{f-\frac{v}{r}-\frac{\partial v}{\partial n}}{d}=const.$$ (11)

The PV conservation law expressed by Eq (10) or (11) allows us to investigate curvature (i.e., topographic) effects in a simplified setting.

## Discussion

In seeking to describe the dynamics of an upwelling-induced coastal current by means of Eq (10) or (11) we can envision: (i) an upwelling along the southern Sicilian coats due to Mistral nortwesterly wind, (ii) the consequent eastward motion of the upwelled current due to the cross-shore pressure gradient (S4 Fig), and (iii) the coastal flow response to the topographic effects, in particular, between Malta and the Sicilian coast, where seafloor curvature is ≠ 0 (Fig 1).

For a “narrow” coastal flow (with *W*/*L* << 1), which is formed by a weak upwelling, Eq (11) shows that, as the flow thickness *d* tends to decrease because of a decrease of the sea bottom—i.e., a topographic curvature toward the right (i.e. offshore) as occurring between Malta and the Sicilian coast (Fig 1)—the numerator of (11) must decrease as well. This means that the current, which can be supposed to flow initially along a straight coast (i.e., *r* ~ ∞) will increase its curvature in order to flow, geostrophically, along the isobaths. Accordingly, a stretching of *d* in Eq (11), which corresponds to a topographic curvature towards the left (i.e., onshore), will bring to an increase of the flow curvature and thus to a cyclonic path between Malta and Capo Passero. Such a geostrophic condition, resulting from the “narrow flow” approximation, guarantees that the flow does not separate from the coastal area and the isobaths. From a different prospective, this also implies that a weak (although non-zero) upwelling condition that generates a narrow coastal flow is prone to deliver anchovy larvae to the nursery area off Capo Passero through a coastal geostrophic path.

On the contrary, if upwelling is particularly strong, as for the case in Fig 6, where the SST cold coastal patch is seen to be *W* ≅ 60 km wide, with a the south Sicily coast length being *L* ≅ 240 km, thus yielding *W/L* = O(0.25), then Eq (10) applies in that the advective terms in Eq (2) are no longer negligible. Hence, this brings to the presence of a non-linear local rotation ($\frac{\partial u}{\partial s}-\frac{\partial v}{\partial n}$) that occurs when PV in Eq (10) needs to remain constant: when the flow thickness *d* tends to decrease because of the topographic curvature toward the right, along with an increase of the flow curvature (i.e., *v*/*r*) there is also the increase of negative (clockwise) circulation given by the rotational terms $\frac{\partial u}{\partial s}-\frac{\partial v}{\partial n}$. This results in the formation of an anti-cyclonic eddy that triggers a cross-shore transport.

Such a particular condition can be observed in our results: (i) the Mistral wind produces a significant upwelling off Sciacca (Fig 6 and S3 Fig and see also Fig 1) that, consequently, (ii) triggers a wide, upwelling-induced coastal current at the end of June 2004 (Fig 5; S1 Movie); (iii) the simulated coastal flow separates from the isobaths upstream Malta during the same period (Fig 5; S1 Movie; Eq 10). This non-linear behaviour of the flow is then reasonably responsible for the unexpected offshore branch that flows southwards anti-cyclonically towards Lampedusa and delivers part of the anchovy larvae offshore (Fig 2). We finally remark that the weakening of the Mistral wind reduces the current width and thus re-establishes the alongshore pathway of the coastal, upwelling-induced current (S5 Fig).

All this clearly stress the tight link between biological and physical transport processes in the oceans when, in particular, ocean currents have a range of impacts across taxa. Similar findings are indeed discussed in previous works regarding the role of advection on sea turtle hatchlings and adults in crossing the Mediterranean Sea [16], [59] as well as in studies regarding the connectivity among different benthic species such as sea grasses [60].

## Conclusions

Both large and mesoscale oceanographic patterns in the Sicily Channel are strongly related to the combining effects of wind and seafloor topography. Not surprisingly we here found that wind-induced coastal upwelling along the southern Sicilian coast also impacts the fate and distribution of European anchovy larvae within the Channel. For the case study of the 2004, we argue—by means of experimental and theoretical findings—that a particularly intense upwelling occurring around the beginning of July generated a wide coastal current that gave rise to a non-linear instability: an eddy formation off the island of Malta, where the flow is mainly subjected to a topographic forcing. This sort of flow separation consequently represented an offshore transport of anchovy larvae, which were deflected from their main coastal path that would deliver them to the recruiting area off Capo Passero, the southeastern tip of Sicily. The multivariate EOF analysis clearly distinguishes this retention zone from the offshore sector off the island of Lampedusa by associating negative Chl and SST anomalies and positive KE to the cross-shore transported larvae.

The main mechanism that seems to rule the transport dynamics of anchovy larvae in the Sicily Channel is therefore given by the presence of moderate upwelling that generates a coastal conveyor belt able to efficiently deliver larvae to the recruiting area. However, if upwelling increases and, consequently, the coastal current becomes too wide (i.e., large outcrop of the upwelled isoplycnals) with respect to the topographic curvature, flow instabilities arise and generate flow separation that, in turn, transport anchovy larvae offshore. Finally the abatement of the northwesterly Mistral wind responsible for the upwelling turns the coastal transport off and causes an interruption of the alongshore pathway.

All this highlights the potential of remote sensing techniques to monitor and statistically predict the abundance of anchovy larvae in the main recruiting area off Capo Passero. Indeed the estimation of upwelling indices that account for both wind persistence and SST gradients along the Sicilian southern coast may work as a predictive tool for estimating the efficiency of the coastal conveyor belt in safely guaranteeing the presence of anchovy juveniles in the nursery areas. This should be complemented by an estimation of rate of mortality induced by the mesoscale activity. Such a goal is particular achievable for anchovies, since their larvae are prone to die if experiencing non-favourable sites. This research thus aims to the achievement of a decision support system for the sustainable management of fisheries in the southern regions of Italy, a service that may “connect the dots” between real-time observations (mostly from remote sensing), numerical modelling, and fisheries.

## Supporting Information

S1 Fig Anchovy egg and larvae concentrations. Spatial distribution of anchovy egg (A) and larvae (B) concentrations superimposed on the cruise averaged SST. Triangles in (B) show mean ages of the sampled larvae as in Fig 2.
(PDF)

S2 Fig Percentage of variance explained by each EOF mode. The percentage is computed both in terms of the total variance associated with the normalized multivariate vector and as the percentage of variance explained for each one of the variables that are included in the multivariate analysis. This plot shows that Larvae distribution is mostly described by EOF mode 4 and 5.
(PDF)

S3 Fig Upwelling evolution along the southern Sicilian coast (25-27-29 June 2004). (A, B, C) Satellite-derived sea surface winds and (D, E, F) SST showing the formation of strong upwelling during the end of June 2004 and the consequent formation of a coastal, cold, upwelled current (see also Fig 6 and S5 Fig and S1 Movie). Triangles in panel (D)-(F) show mean larvae ages as in Fig 2. Note that in (A, B, C) and (D, E, F) both sea surface wind and SST palette are different, respectively, for each day in order to stress gradients and patterns.
(PDF)

S4 Fig Upwelled current in a coast-following coordinate system. (A) Curvilinear coordinate system in which *s* and *n* representing the along-shore and cross-shore directions, *r*(*s*) is the radius of curvature of the coastline or bathymetry and D is the (constant) radius of the cylindrical system. (B) schematic representation of the upwelling-induced geostrophic current along the southern Sicilian coast, flowing southeastwards (into the paper) (modified from [58]).
(PDF)

S5 Fig Different patterns for coastal transport. (A, B, C) Satellite-derived Ekman transport maps; (D, E, F) high resolution MODIS SST non-interpolated satellite maps; (G, H, I) snapshot of the Lagrangian runs [35] as in Fig 6, where the blue arrows represent the main path(s) of the upwelling-induced coastal current. The figure shows three different scenarios regarding the coastal transport due to the onset and withdrawal of the upwelling-induced coastal current: (A, D, G) cross-shore transport due to non-linear instabilities that are triggered by a wide coastal current and strong upwelling (18 June 2004); (B, E, H) efficient coastal transport with no instabilities due to a narrow coastal flow and mild upwelling (1 September 2004); (C, F, I) withdrawal of the upwelled current (from 2 September 2004).
(PDF)

S1 Movie Lagrangian transport of anchovy larvae. Animated gif showing the Lagrangian transport of tracers. The simulation starts from 1 June 2004 and last for 120 days until the end of September (after [35]). Tracers' colors indicate larvae age as in Fig 6.
(GIF)

S1 Table List of satellite products. *Δt* and *Δx* indicate temporal and spatial resolution of the data. SST: sea surface temperature L4 multi-sensor and MODIS L3; Chl: surface chlorophyll from SeaWiFS; Kd490: diffuse light attenuation coefficient, at 490 nm from SeaWiFS; Ocean Wind: Cross-Calibrated, Multi-Platform Ocean Surface Wind Velocity Product (multi-sensor, made of SeaWinds su QuikSCAT e ADEOS-II, AMSR-E, TRMM TMI, SSM/I); Sea surface geostrophic velocity: multimission altimeter products (Saral, Cryosat-2, Jason-1&2, T/P, Envisat, GFO, ERS-1 & 2 and even Geosat).
(DOC)
