# Correction: The Role of Hydrodynamic Processes on Anchovy Eggs and Larvae Distribution in the Sicily Channel (Mediterranean Sea): A Case Study for the 2004 Data Set

**DOI:** 10.1371/journal.pone.0129990

**Published:** 2015-06-09

**Authors:** The PLOS ONE Staff

Fig 6 incorrectly appears as a duplicate of Fig 5. The authors have provided a corrected version here.

**Fig 6:**
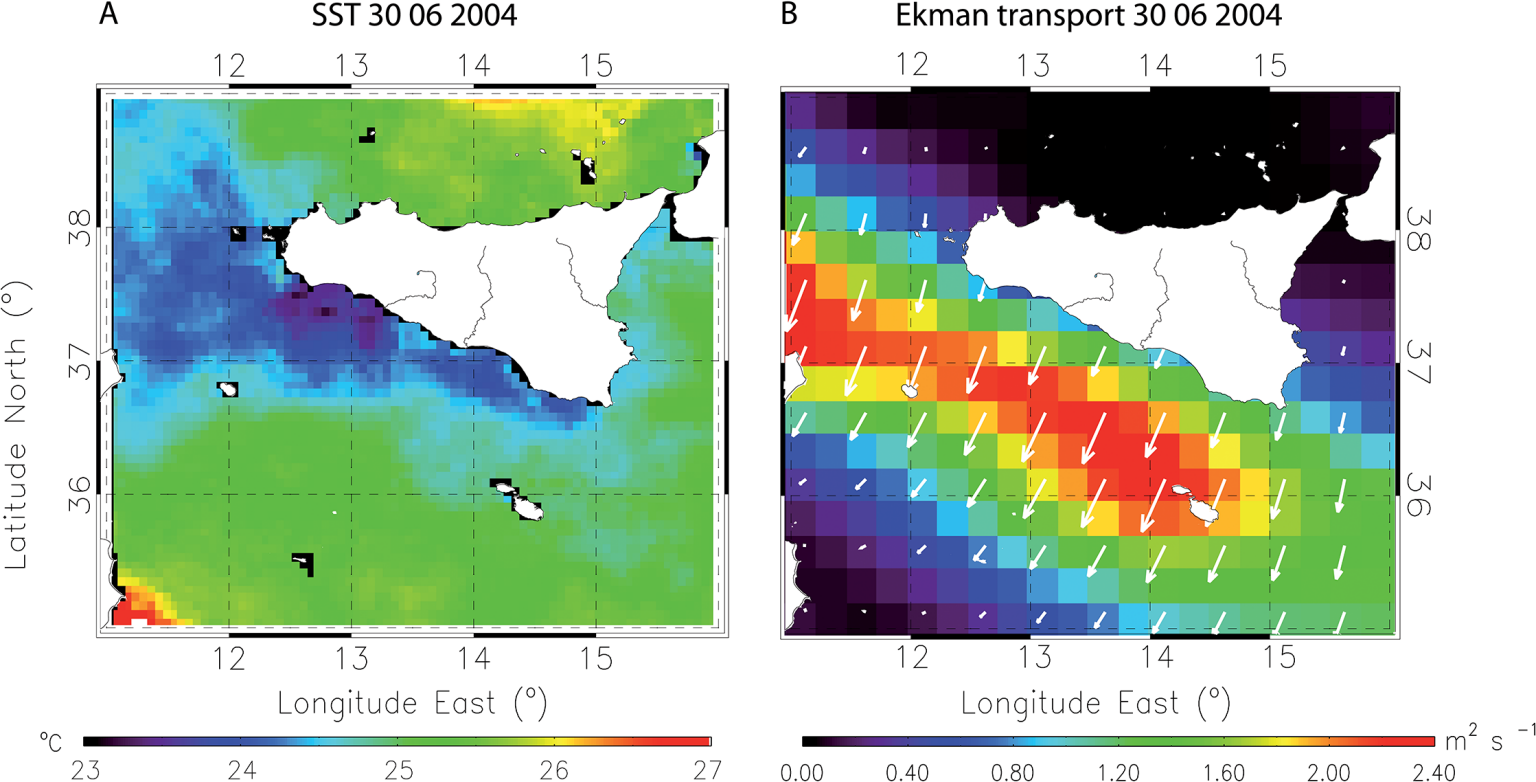
Coastal upwelling along the Sicilian coast (30 June 2004). It shows the formation of an upwelled coastal pattern marked by (A) a strong satellite SST gradient along the southern Sicilian shoreline and (B) a strong Ekman offshore transport (see also S2 Fig).
