# Supplementary figures and images for: The Role of Hydrodynamic Processes on Anchovy Eggs and Larvae Distribution in the Sicily Channel (Mediterranean Sea): A Case Study for the 2004 Data Set
Source: PLoS One. 2015 Apr 27;10(4):e0123213. doi: 10.1371/journal.pone.0123213 (PMC4411133; doi:10.1371/journal.pone.0123213)

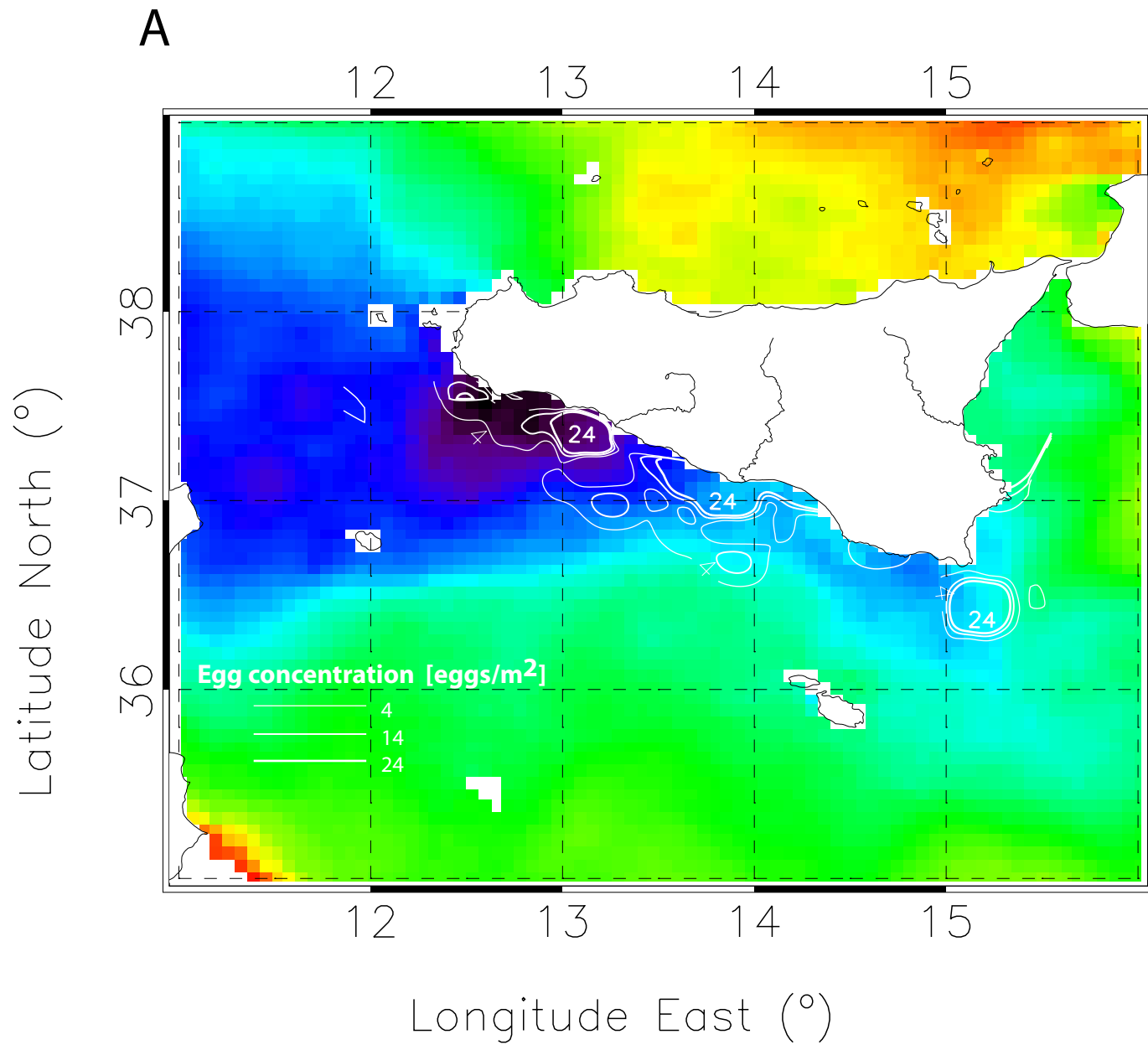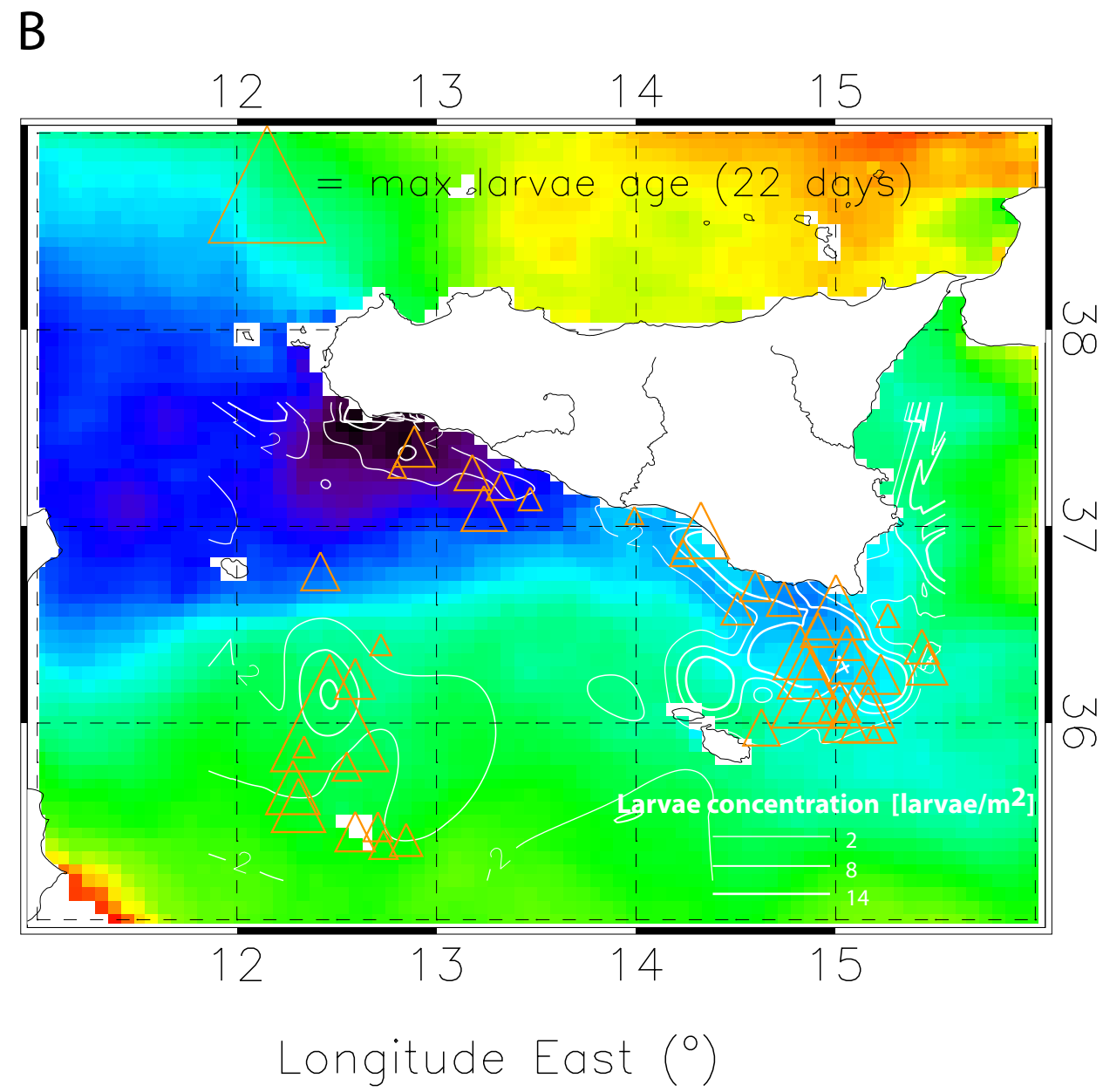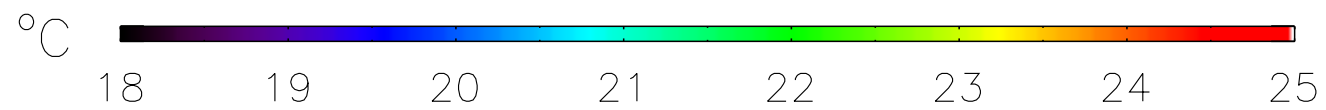

Supplement: S1 Fig — Spatial distribution of anchovy egg (A) and larvae (B) concentrations superimposed on the cruise averaged SST. Triangles in (B) show mean ages of the sampled larvae as in Fig 2. (PDF) [file pone.0123213.s001.pdf]

variance explained by single EOF mode

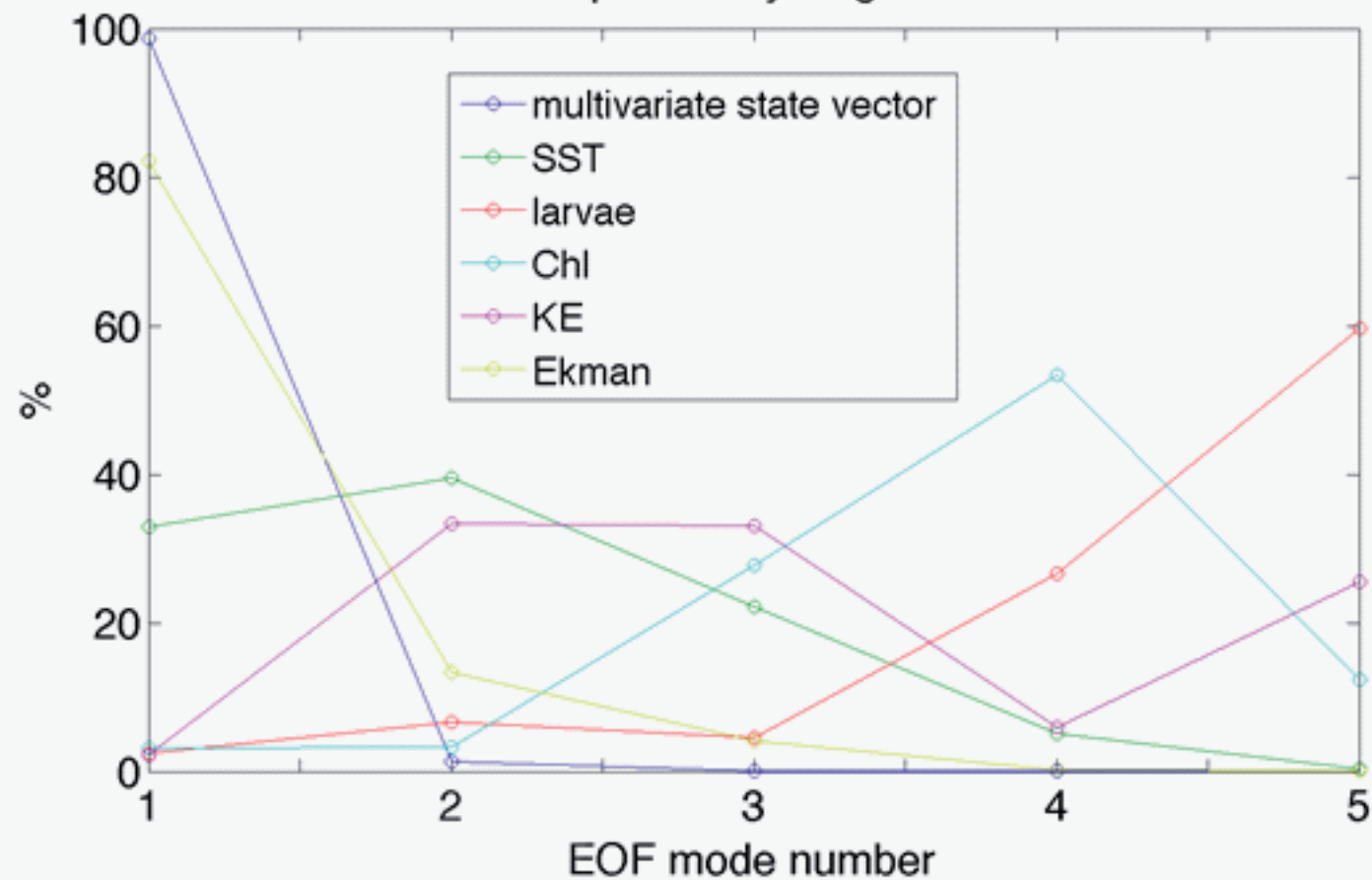

Supplement: S2 Fig — The percentage is computed both in terms of the total variance associated with the normalized multivariate vector and as the percentage of variance explained for each one of the variables that are included in the multivariate analysis. This plot shows that Larvae distribution is mostly described by EOF mode 4 and 5. (PDF) [file pone.0123213.s002.pdf]

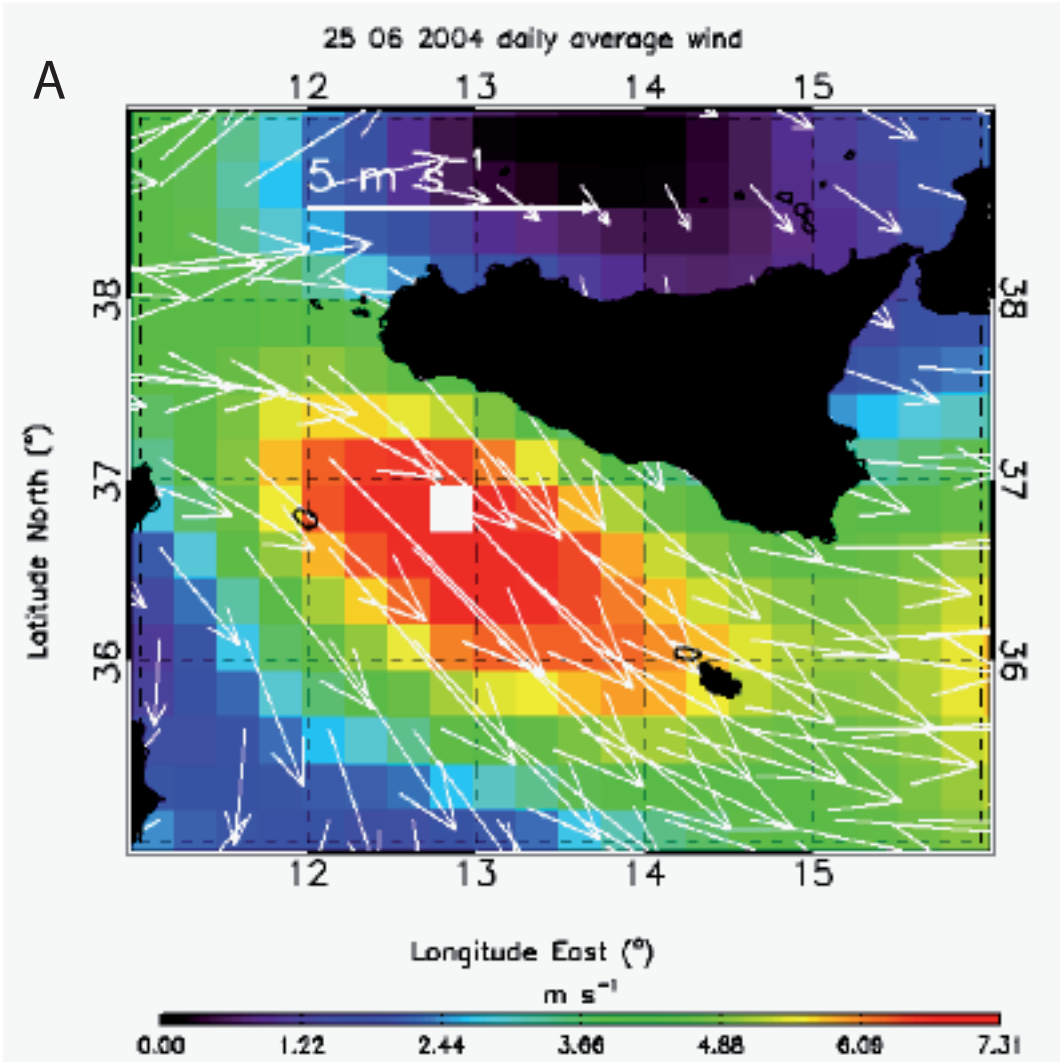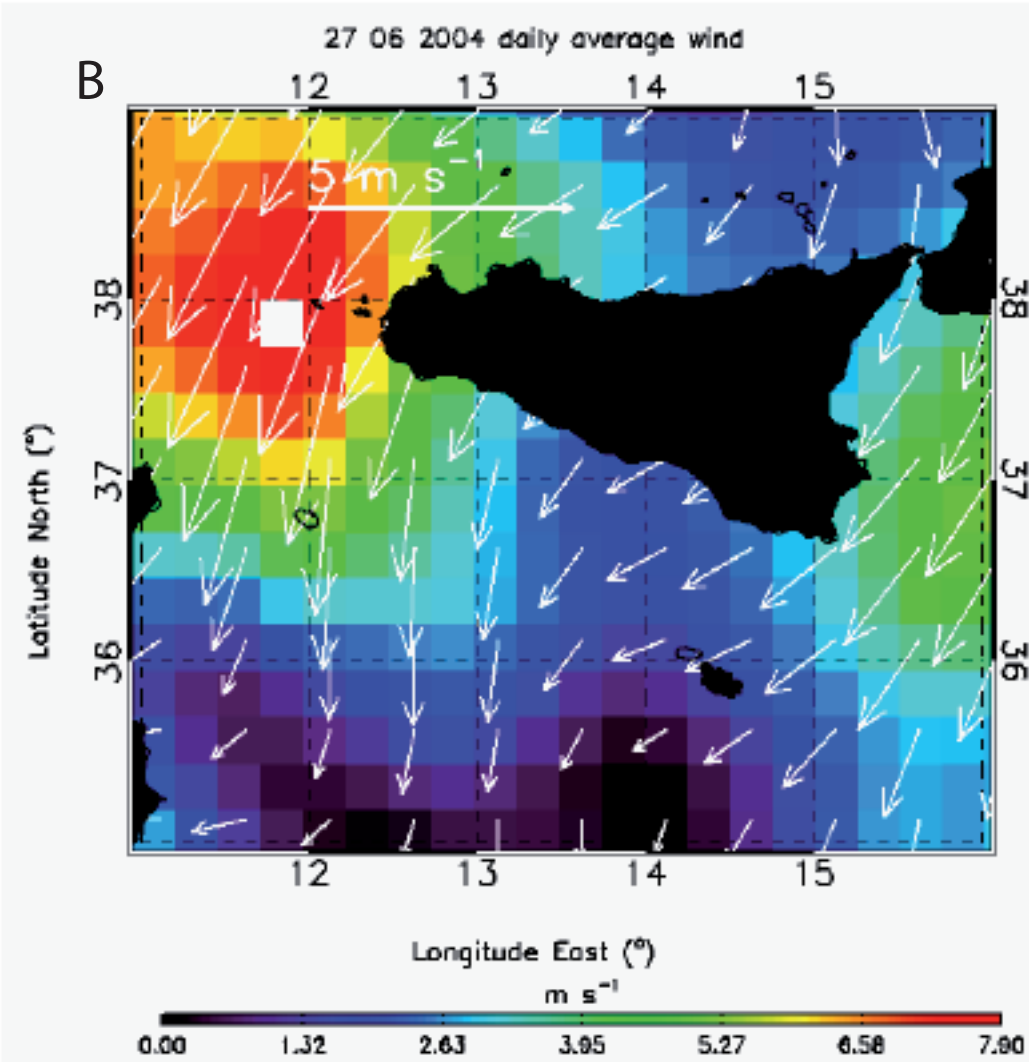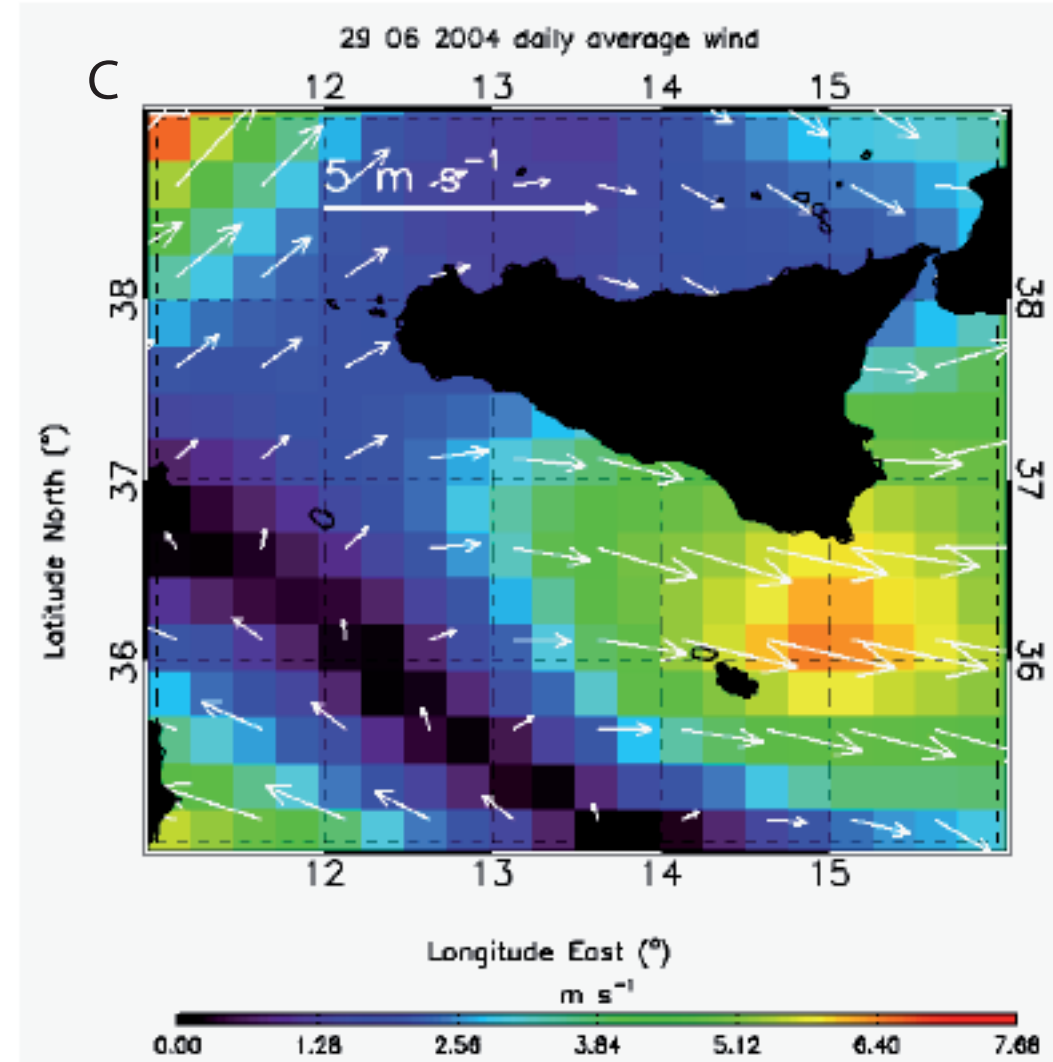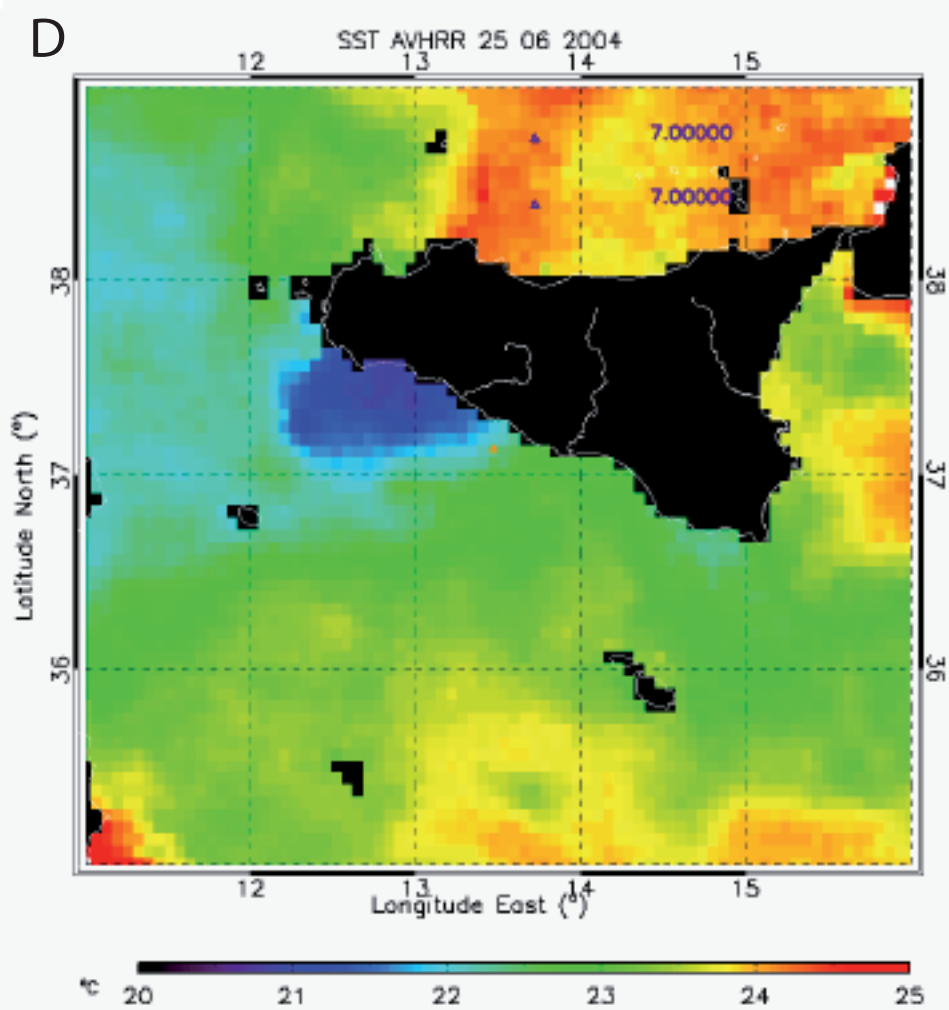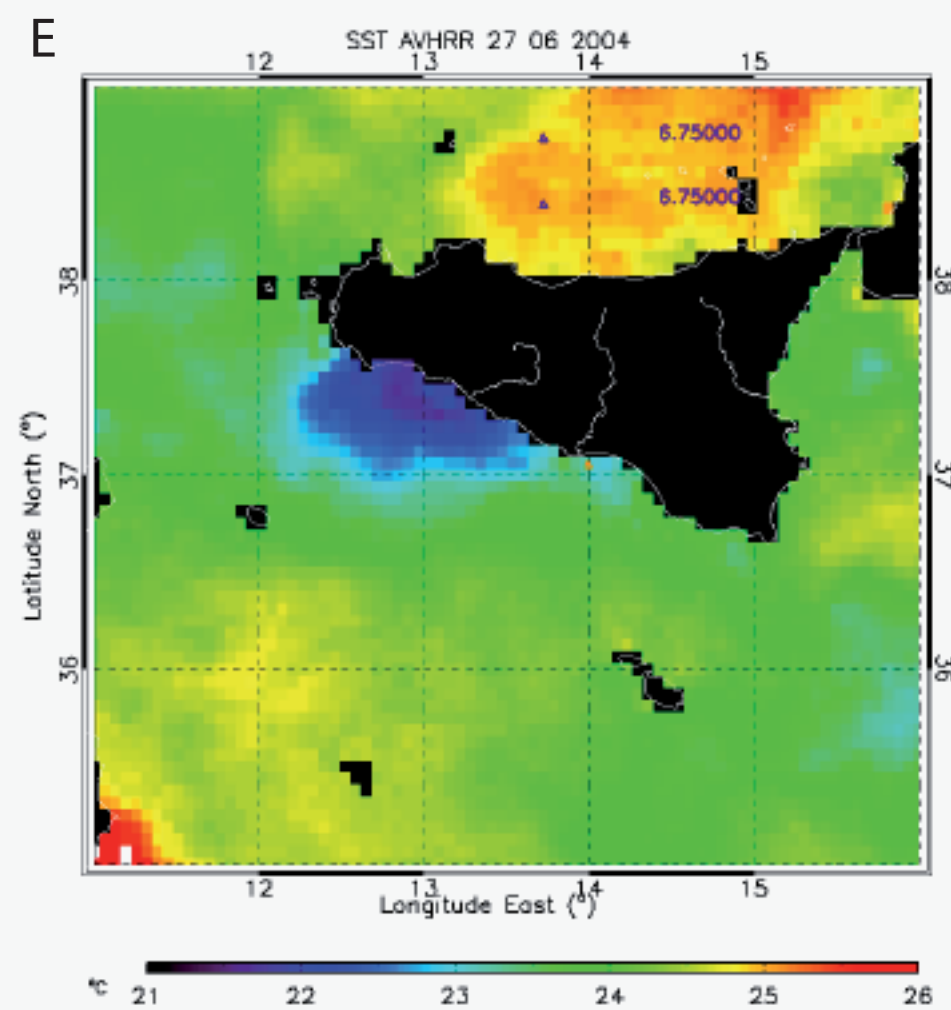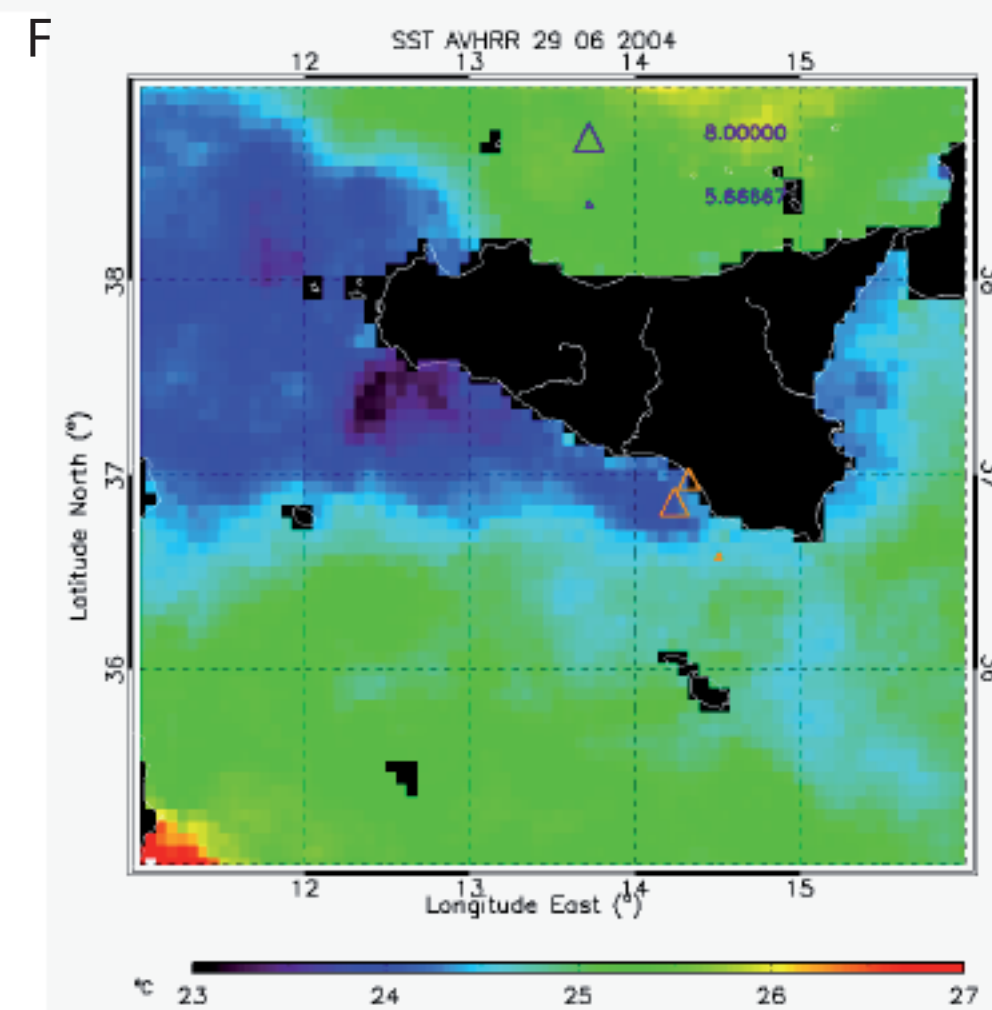

Supplement: S3 Fig — (A, B, C) Satellite-derived sea surface winds and (D, E, F) SST showing the formation of strong upwelling during the end of June 2004 and the consequent formation of a coastal, cold, upwelled current (see also Fig 6 and S5 Fig and S1 Movie). Triangles in panel (D)-(F) show mean larvae ages as in Fig 2. Note that in (A, B, C) and (D, E, F) both sea surface wind and SST palette are different, respectively, for each day in order to stress gradients and patterns. (PDF) [file pone.0123213.s003.pdf]

A

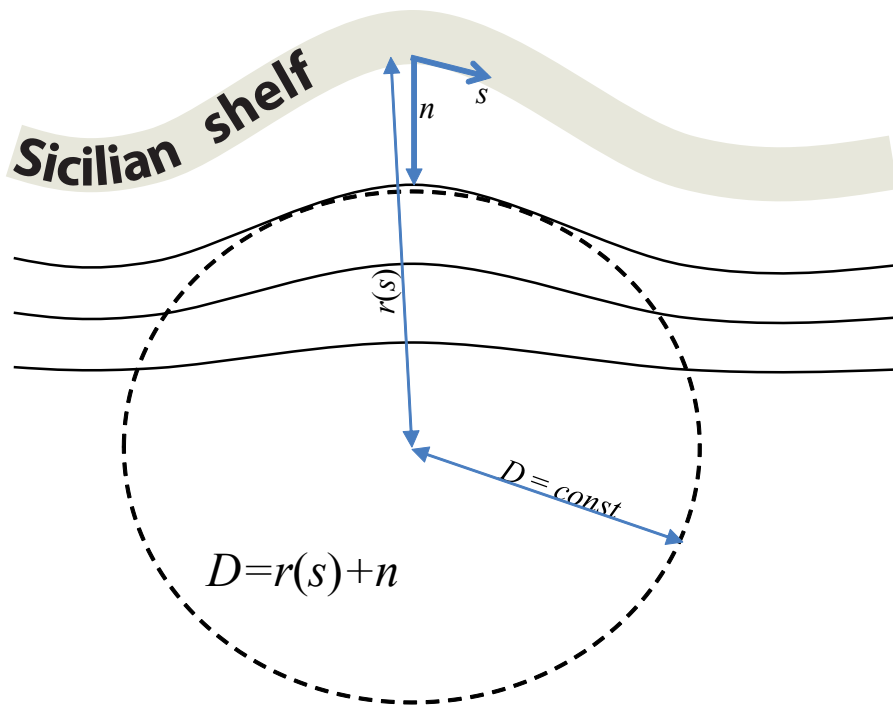

B

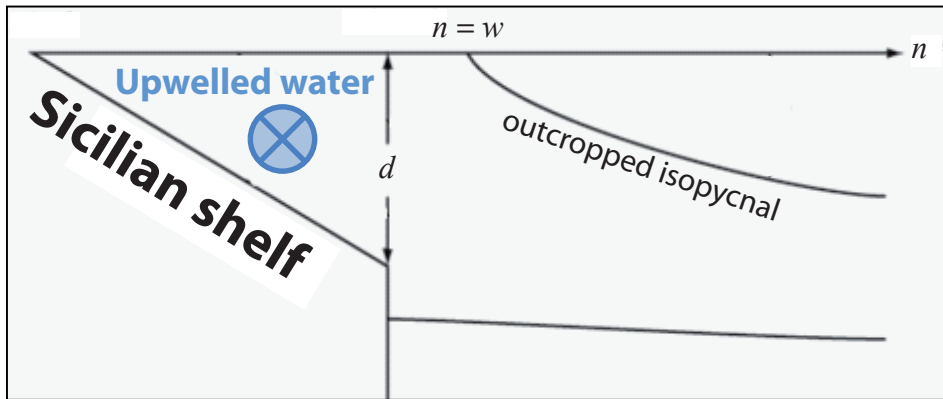

Supplement: S4 Fig — (A) Curvilinear coordinate system in which s and n representing the along-shore and cross-shore directions, r(s) is the radius of curvature of the coastline or bathymetry and D is the (constant) radius of the cylindrical system. (B) schematic representation of the upwelling-induced geostrophic current along the southern Sicilian coast, flowing southeastwards (into the paper) (modified from [58]). (PDF) [file pone.0123213.s004.pdf]

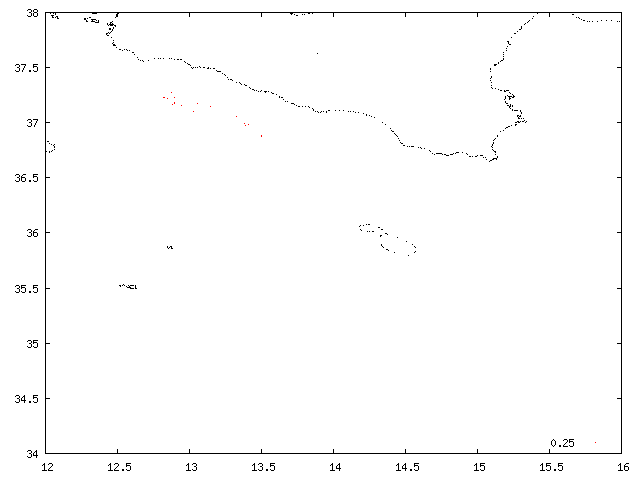

Supplement: S1 Movie — Animated gif showing the Lagrangian transport of tracers. The simulation starts from 1 June 2004 and last for 120 days until the end of September (after [35]). Tracers' colors indicate larvae age as in Fig 6. (GIF) [file pone.0123213.s006.gif]
