# Supplementary material for: The Role of Hydrodynamic Processes on Anchovy Eggs and Larvae Distribution in the Sicily Channel (Mediterranean Sea): A Case Study for the 2004 Data Set
Source: PLoS One. 2015 Apr 27;10(4):e0123213. doi: 10.1371/journal.pone.0123213 (PMC4411133; doi:10.1371/journal.pone.0123213)
Supplement: S1 Table — Δt and Δx indicate temporal and spatial resolution of the data. SST: sea surface temperature L4 multi-sensor and MODIS L3; Chl: surface chlorophyll from SeaWiFS; Kd490: diffuse light attenuation coefficient, at 490 nm from SeaWiFS; Ocean Wind: Cross-Calibrated, Multi-Platform Ocean Surface Wind Velocity Product (multi-sensor, made of SeaWinds su QuikSCAT e ADEOS-II, AMSR-E, TRMM TMI, SSM/I); Sea surface geostrophic velocity: multimission altimeter products (Saral, Cryosat-2, Jason-1&2, T/P, Envisat, GFO, ERS-1 & 2 and even Geosat). (DOC) [file pone.0123213.s007.doc]

| Parameter | *Δt* | *Δx* | Data source |
| --- | --- | --- | --- |
| SST L4 | daily | 7 × 7 km | http://gosweb.artov.isac.cnr.it/ |
| SST L3 | daily | 1 × 1 km | http://oceancolor.gsfc.nasa.gov |
| Chl | daily | 1 × 1 km | <http://www.myocean.eu.org/>, http://gosweb.artov.isac.cnr.it/ |
| Kd490 | daily | 1 × 1 km | <http://www.myocean.eu.org/>, http://gosweb.artov.isac.cnr.it/ |
| Ocean Wind | daily | 25 × 25 km | http://podaac.jpl.nasa.gov |
| Sea surface geostrophic velocity | daily | 25 × 25 km | <http://www.myocean.eu.org/>, http://www.aviso.altimetry.fr |

**Table S1**. List of satellite products. *Δt* and *Δx* indicate temporal and spatial resolution of the data. SST: sea surface temperature L4 multi-sensor and MODIS L3; Chl: surface chlorophyll from SeaWiFS; Kd490: diffuse light attenuation coefficient, at 490 nm from SeaWiFS; Ocean Wind: Cross-Calibrated, Multi-Platform Ocean Surface Wind Velocity Product (multi-sensor, made of SeaWinds su QuikSCAT e ADEOS-II, AMSR-E, TRMM TMI, SSM/I); Sea surface geostrophic velocity: multimission altimeter products (Saral, Cryosat-2, Jason-1&2, T/P, Envisat, GFO, ERS-1 & 2 and even Geosat).
