# Supplementary material for: The Role of Hydrodynamic Processes on Anchovy Eggs and Larvae Distribution in the Sicily Channel (Mediterranean Sea): A Case Study for the 2004 Data Set
Source: PLoS One. 2015 Apr 27;10(4):e0123213. doi: 10.1371/journal.pone.0123213 (PMC4411133; doi:10.1371/journal.pone.0123213)
Supplement: S5 Fig — (A, B, C) Satellite-derived Ekman transport maps; (D, E, F) high resolution MODIS SST non-interpolated satellite maps; (G, H, I) snapshot of the Lagrangian runs [35] as in Fig 6, where the blue arrows represent the main path(s) of the upwelling-induced coastal current. The figure shows three different scenarios regarding the coastal transport due to the onset and withdrawal of the upwelling-induced coastal current: (A, D, G) cross-shore transport due to non-linear instabilities that are triggered by a wide coastal current and strong upwelling (18 June 2004); (B, E, H) efficient coastal transport with no instabilities due to a narrow coastal flow and mild upwelling (1 September 2004); (C, F, I) withdrawal of the upwelled current (from 2 September 2004). (PDF) [file pone.0123213.s005.pdf]

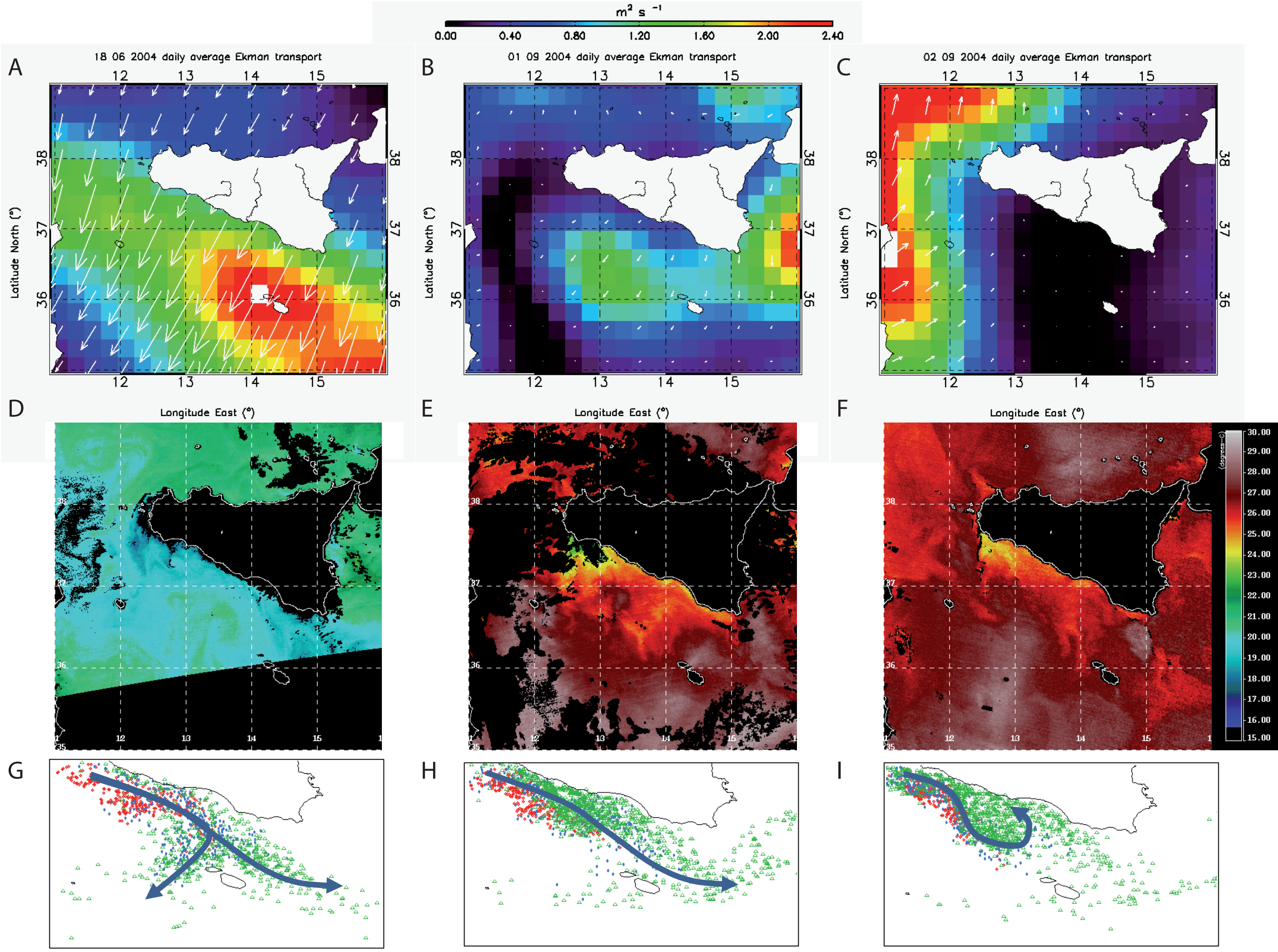

m² s⁻¹
0.00 0.40 0.80 1.20 1.60 2.00 2.40
A
18 06 2004 daily average Ekman transport
B
01 09 2004 daily average Ekman transport
C
02 09 2004 daily average Ekman transport
Latitude North (°)
Longitude East (°)
D
E
F
G
H
I
30.00 29.00 28.00 27.00 26.00 25.00 24.00 23.00 22.00 21.00 20.00 19.00 18.00 17.00 16.00 15.00
